# Putative linkages between below- and aboveground mutualisms during alien plant invasions

**DOI:** 10.1093/aobpla/plv062

**Published:** 2015-06-01

**Authors:** Susana Rodríguez-Echeverría, Anna Traveset

**Affiliations:** 1 CFE-Centre for Functional Ecology, Department of Life Sciences, University of Coimbra, 3000-456 Coimbra, Portugal.; 2 Mediterranean Institute of Advanced Studies (CSIC-UIB), C/Miquel Marqués 21, E07190 Esporles, Mallorca, Spain

**Keywords:** Community dynamics, invasion, mutualism, mycorrhiza, pollination, rhizobia, seed dispersal, symbiosis

## Abstract

Evidence of the fundamental role of below–aboveground links in controlling ecosystem processes is mostly based on studies done with soil herbivores or mutualists and aboveground herbivores. Much less is known about the links between belowground and aboveground mutualisms, which have been studied separately for decades. It has not been until recently that these mutualisms—mycorrhizas and legume–rhizobia on one hand, and pollinators and seed dispersers on the other hand—have been found to influence each other, with potential ecological and evolutionary consequences. Here we review the mechanisms that may link these two-level mutualisms, mostly reported for native plant species, and make predictions about their relevance during alien plant invasions. We propose that alien plants establishing effective mutualisms with belowground microbes might improve their reproductive success through positive interactions between those mutualists and pollinators and seed dispersers. On the other hand, changes in the abundance and diversity of soil mutualists induced by invasion can also interfere with below–aboveground links for native plant species. We conclude that further research on this topic is needed in the field of invasion ecology as it can provide interesting clues on synergistic interactions and invasional meltdowns during alien plant invasions.

## Abstract (precis)

Plants establish positive interactions with many other species that allow them to improve their chances of establishing, surviving, growing and reproducing successfully. Positive interactions, or mutualisms, involve both bacteria and fungi in the soil and aboveground animals that pollinate or disperse plant seeds. Until very recently, these mutualisms have been studied separately but there is evidence that they can influence each other and, thus, plant fitness and the dynamics of plant populations. This review explores links between these mutualists during plant invasion and their relevance to understanding this process and managing exotic plant species more effectively.

## Introduction

Interactions and feedbacks between belowground and aboveground subsystems play a fundamental role in regulating community structure and ecosystem functioning (e.g. Bever *et al*. 1997, 2010, 2012; Wardle *et al*. 2004; Kulmatiski *et al*. 2008; Bardgett and Wardle 2010; Bezemer *et al*. 2013; Fukami and Nakajima 2013; Kardol *et al*. 2013; Van der Putten *et al*. 2013; Bardgett and van der Putten 2014). However, studies examining below–aboveground interactions have focussed mainly on below- and aboveground herbivores (e.g. Moran and Whitham 1990; Bezemer and van Dam 2005; Ruijven *et al*. 2005; Mckenzie *et al*. 2013; de la Peña and Bonte 2014) or, to a lesser extent, on belowground mutualists and aboveground herbivores (e.g. Gehring and Whitham 1994, 2002; Gange *et al*. 2002; Kempel *et al*. 2009; Koricheva *et al*. 2009; Katayama *et al*. 2011; Thamer *et al*. 2011; Babikova *et al*. 2014). The interaction between belowground mutualists and aboveground endophytes has been studied in invasive and non-invasive plants, showing that both microbial groups can affect each other and have synergistic effects on the host plant (e.g. Omacini *et al*. 2006; Larimer *et al*. 2010, 2012; García-Parisi *et al*. 2015). Much less is known about the links connecting belowground and aboveground mutualists involved in plant reproduction. These two types of mutualisms have been studied for decades, but separately, and it has not been until recently that they have been documented to influence each other. A few studies have shown the influence of mycorrhizas on reproductive traits and pollinators, indicating that these unexplored interactions can have both ecological and evolutionary consequences.

Research during the last decade has also shown that plant–soil feedbacks might determine the invasive success of many alien plant species (e.g. Klironomos 2002; de la Peña *et al*. 2010; Inderjit and van der Putten 2010; Zhang *et al*. 2010; Callaway *et al*. 2011; Suding *et al*. 2013; but see Birnbaum and Leishman 2013). However, to our knowledge, linkages between belowground and aboveground mutualisms have received no attention when studying alien plant invasions. Investigating these putative links might provide interesting clues on synergistic interactions and invasional meltdown processes during plant invasions.

The main goal of this review is, thus, to identify the different mechanisms by which below- and aboveground mutualisms can be linked as well as the different outcomes regarding the spread of the invasive plants involved in such mutualisms. It is not our intention here either to analyse how different types of mutualisms enhance invasions or the impact of invaders on particular interactions, as these have been explored in recent reviews (e.g. Pringle *et al*. 2009*b*; Kiers *et al*. 2010; Hale and Kalisz 2012; Nuñez and Dickie 2014; Traveset and Richardson 2014). Instead, we review studies that have linked both types of mutualisms in the context of community ecology and predict how these below–aboveground linkages between mutualisms could affect plant invasions.

## Below- and Aboveground Plant Mutualisms

Regarding mutualistic soil microorganisms, we will focus on mycorrhizas (associations between soil fungi and roots of vascular plants) and rhizobia (α- or β-Proteobacteria engaged in symbiosis with legumes). Mycorrhizas occur in more than 90 % of the examined plant families (Brundrett 2009), and at least in a third of the world's most widespread invasive woody species (Traveset and Richardson 2014). The two main types of mycorrhizas are arbuscular mycorrhizas, occurring in 80 % of plant families, from grasslands to tropical forests; and ectomycorrhizas, dominating woodlands and forests in boreal, Mediterranean and temperate areas (Brundrett 2009; Barea *et al*. 2011). The legume–rhizobia symbiosis, on the other hand, is prevalent in most terrestrial ecosystems, occurring in ∼80 % of legumes, and is responsible for over 90 % of the biologically fixed nitrogen entering into terrestrial ecosystems worldwide (Sprent 2001). Both types of belowground mutualisms are crucial for the uptake of nutrients and water by plants, and, as such, can play an important role in determining the structure and dynamic of terrestrial ecosystems (e.g. Wall and Moore 1999; van der Heijden 2002; van der Heijden *et al*. 2006; Klironomos *et al*. 2011). However, the degree of dependency on soil mutualisms varies for different plant species and the net benefit for the plant can also change depending on the plant species, mycorrhizal type and biotic conditions (Johnson *et al*. 1997; Pringle *et al*. 2009*b*; Hoeksema *et al*. 2010; Thrall *et al*. 2011). Nonetheless, most of these studies have been done using inoculation experiments in the greenhouse and this could not reflect the real outcome of these mutualisms in the field (Hoeksema *et al*. 2010).

Aboveground, we will be centred on the most conspicuous mutualisms involving plants, *viz.* pollination and seed dispersal. These mutualisms are directly associated with the reproduction of plant species and, thus, with their ability to establish self-replacing populations. Therefore, these two types of mutualisms are crucial to understand the spatial structure and demographic processes of populations and communities (Bond 1994). Although wind pollination is common among monocots, biotic pollination is an essential ecosystem service as >90 % of flowering plants are pollinated by animals (Ollerton *et al*. 2011) and ∼75 % of the world's main food crops are obligate out-crossers (Klein *et al*. 2007). Animals also disperse up to 90 and 60 % of plant species in tropical and temperate regions, respectively (Farwig and Berens 2012), and their service provides a way to escape from competing siblings and natural enemies around parent plants at the same time that facilitates the colonization of vacant recruitment sites. Moreover, such service helps in maintaining genetic diversity and drives adaptation of plants to changing environments (Traveset *et al*. 2013). Most flowering and fleshy-fruited plants rely on generalist pollinators/dispersers, i.e. animals that pollinate/disperse a wide range of plants (Ollerton *et al*. 2011; Farwig and Berens 2012; Traveset *et al*. 2013). This indeed facilitates the integration of alien plants and alien pollinators/dispersers into the native pollination/disperser networks (Stouffer *et al*. 2014; Traveset and Richardson 2014).

Belowground mutualists may affect not only plant growth but also reproductive success through changes in flower display and/or fruit display. The quantity and quality of pollen, nectar and fruit pulp are directly related to the plant nutritional status, and serve as attractive cues for pollinators and seed dispersers. Therefore, alterations in those traits due to the presence of belowground mutualisms are likely to affect aboveground mutualisms. In the next sections we go in depth into these mechanisms. We focus on the effect of belowground on aboveground mutualisms, as this is what has been mostly studied. However, the reciprocal effect must also be prevalent, at least indirectly, as pollinators and dispersers obviously influence plant population density and structure and this, in turn, has a direct effect on the community of microorganisms belowground.

## Effect of Belowground Mutualisms on Plant Reproductive Traits and Aboveground Mutualisms

Most research on the effect of belowground mutualists on plant reproductive traits and reproductive output has been done using arbuscular mycorrhizal (AM) fungi and annual plants (Table 1). For such interactions, there is robust evidence on the positive effect of mycorrhization in reproduction, most likely driven by the improved plant phosphorus uptake (Fitter 1990; Koide and Dickie 2002; Koide 2010).

**Table 1.** Effect of mycorrhizal fungi on reproductive traits and success, and on pollinator visitation rates. The effect of mycorrhizas on pollinators is mediated by their effect on plant reproductive traits, and might have consequences for the quantity and quality of the offspring. Asterisks mean that the effect of mycorrhizas changed for different groups of pollinators.

Plant species	Reproductive traits	Pollination	Offspring production	Seeds and seedlings quality	Authors
*Abutilon theophrasti*	Earlier flowering		Increased fruit set	Increased seed weight	Stanley *et al*. (1993), Heppell *et al*. (1998), Koide *et al*. (1994), Lu and Koide (1994), Shumway and Koide (1994, 1995) and Koide (2010)
	Increased number of flowers per plant		Increased number of seeds per fruit	Increased seed N and P content	
				Increased seedling vigour	
				Increased competitive ability	
*Avena fatua*	No effect on the number of panicles		Increased number of seeds per plant	Increased seed P content	Koide *et al*. (1988), Lu and Koide (1991) and Koide and Lu (1992)
	Decreased flowering time		Increased total seed weight per plant	No effect on seed N content	
				Increased seedling vigour	
*Avena sativa*	Increased number of panicles		Increased number of seeds per plant	Increased seed P content	Koide *et al*. (1988)
	Increased flowering time		Increased total seed weight per plant	No effect on seed N content	
*Calluna vulgaris*		Increased*			de la Peña *et al*. (2012)
*Campanula rotundifolia*	Lower number of flowers			Increased seed P content	Nuortila *et al*. (2004)
				Increased seedling vigour	
*Capsicum annuum*	Increased number of flowers				Dodd *et al*. (1983)
*Centaurea cyanus*	Increased number of flowers	Increased			Gange and Smith (2005)
*Chamerion angustifolium*	Greater floral display	Increased	Increased seed set		Wolfe *et al*. (2005)
*Cucurbita foetidissima*	Increased number of male flowers per plant				Pendleton (2000)
*Cucurbita pepo*	Increased pollen production				Lau *et al*. (1995)
*Geranium sylvaticum*	Increased flower size	Increased*			Varga and Kytöviita (2010)
	Increased pollen production				
*Glycine max*	Increased number of flowers		Increased seed yield		Schenck and Smith (1982) and Vejsadova *et al*. (1993)
			Increased fruit set		
*Hordeum vulgare*			Increased seed yield		Clarke and Mosse (1981), Powell (1981) and Jensen (1982)
*Kummerowia striata*	Earlier flowering				Nakatsubo (1997)
*Lycopersicon esculentum*	Higher number of inflorescences		Increased fruit set	No effect	Bryla and Koide (1990), Poulton *et al*. (2001*a*, *b*, 2002) and Koide (2010)
	Higher number of flowers		Increased number of seeds per fruit		
	Increased pollen production		Increased fruit mass		
	Increased pollen quality				
*Lythrum salicaria*	Increased pollen production				Philip *et al*. (2001)
*Petunia* sp.	Earlier flowering				Daft and Okusanya (1973)
*Polemonium viscosum*	Changes in VOC and reduced nectar sugar content	No effect			Becklin *et al*. (2011)
*Tagetes erecta*	Increased nectar availability	Increased			Gange and Smith (2005)
*Tagetes patula*	Increased flower size	Increased			Gange and Smith (2005)
*Triticum aestivum*				Increased seed weight and P content	Karagiannidis and Hadjisavva-Zinoviadi (1998)
*Vaccinium corymbosum*			Increased fruit yield		Powell and Bates (1981)

About 90 % of the studies on AMF and reproduction in annual plants (*n* = 31) have analysed the effect of mycorrhization on reproductive traits (Table 1). Over 80 % of these studies found a positive effect of AMF on the production of flowers, pollen and/or nectar, whereas 12.5 % showed a negative effect of mycorrhization on these traits. Similarly, 90 % of studies reported an increased duration of flowering, although this has been analysed only for a reduced number of plant species (Table 1). Mycorrhization can change the number and size of flowers produced by a plant as well as the length of flowering, and can also reduce fruit and seed abortion (Lu and Koide 1994; Wolfe *et al*. 2005). Moreover, mycorrhizal colonization has been found to significantly increase the size of pollen grains, the total amount of pollen per flower (Lau *et al*. 1995) and *in vitro* pollen tube growth rate (Poulton *et al*. 2001*a*, *b*), which might allow pollen produced by mycorrhizal plants to outcompete slower-growing pollen from non-mycorrhizal plants.

Changes in reproductive traits due to mycorrhization can provide an interesting link between belowground (mutualistic fungi) and aboveground mutualists since these traits—flower size, number, etc.—can be used as attractive cues by pollinators. Wolfe *et al*. (2005) provided the first experimental support for a link between mycorrhizas and pollinators. These authors demonstrated in a field experiment that AM fungi lead to a 2-fold increase in pollinator visitation rate and seed set of *Chamerion angustifolium* (L.) Holub. The effect of AM fungi on pollinators was mediated by a greater floral display due to mycorrhizal plants being taller and having more flowers. Interestingly, the larger size of mycorrhizal plants did not attract more phytophagous insects (Wolfe *et al*. 2005). Increased pollinator visitation rates for mycorrhizal plants have been subsequently reported for other species (Table 1). These studies show that the main mechanisms by which mycorrhizas affect pollinators—increased number of flowers, increased flower size or changing pollen and nectar quantity and quality—vary among plant species (Gange and Smith 2005; Cahill *et al*. 2008; Varga and Kytöviita 2010; Becklin *et al*. 2011). AM colonization also modifies the emission of volatile organic compounds, thus altering floral fragrance and plant attractiveness to pollinators (Becklin *et al*. 2011).

Although mycorrhizal plants have an overall increase in pollinator visitation rates, not all insects respond equally to the presence of AM fungi (Table 1), which has ecological and evolutionary consequences. The suppression of mycorrhizal fungi has been found to change the community of floral visitors in grasslands from large-bodied bees to small-bodied bees and flies, and to reduce the total number of floral visits per flowering stem 67 % across 23 flowering species (Cahill *et al*. 2008). In contrast, other studies show a greater effect of mycorrhizas on hymenopterans and syrphid flies (Gange and Smith 2005; Varga and Kytöviita 2010). Since the interaction between arbuscular mycorrhizas, plant reproductive traits and pollinators in the field is mediated by the composition of the plant community (Cahill *et al*. 2008) and by abiotic conditions (Becklin *et al*. 2011), different outcomes might be found for different species and sites.

The effect of mycorrhizas on floral traits and pollinator visits has been hypothesized to be stronger in annual than in perennial plants because the latter have more complex physiological tradeoffs and ecological interactions determining reproductive success (Becklin *et al*. 2011). Nevertheless, a positive effect of mycorrhization on pollinator visitation rates and plant choice by honeybees has been observed for the shrub *Calluna vulgaris* (de la Peña *et al*. 2012). Owing to the longer life-span of woody plants and the succession of mycorrhizal fungi in their roots (Twieg *et al*. 2007; Peay *et al*. 2011), the influence of mycorrhizas in reproduction might also change with time. As far as we are aware, however, the study on *C. vulgaris* mentioned above is the only available report exploring this link for mycorrhizal woody plants in natural ecosystems.

Seed production can be significantly enhanced in mycorrhizal plants, due not only to a higher overall plant nutrient content but also to changes in nutrient allocation within the plant (Koide and Dickie 2002; Koide 2010, and references therein). Fruit and seed production consistently appear to be enhanced by mycorrhization in almost all plant species examined so far (Table 1). Not only mycorrhizal plants produce more seeds than non-mycorrhizal plants, but seeds have higher phosphorus content, which has a direct effect on offspring performance (Koide and Dickie 2002). Only one study showed no increase in seed weight by mycorrhization although the number of fruits produced per plant was higher for mycorrhizal plants (Bryla and Koide 1990). Such higher seed quantity and/or quality in mycorrhizal plants is very likely to influence positively dispersal success, as frugivores (either vertebrates or invertebrates) are known to have preferences for larger fruit/seed displays and for higher quality of fruits and seeds (Jordano 2013). To our knowledge, however, no study has examined yet the direct or indirect effect of mutualistic microorganisms on the animal dispersal success of any plant species.

Seedlings produced from mycorrhizal plants also grow faster, recruit better and can outcompete seedlings compared with those from non-mycorrhizal plants, which can have an important effect on plant population demography (Stanley *et al*. 1993). This process can in fact be highly relevant for the establishment and spread of invasive plant species.

The effect of nitrogen-fixing mutualists on plant reproduction and pollination has been less studied. Rhizobia can modify plant functional traits related to plant reproduction (Friesen *et al*. 2011), although few empirical studies have tested this premise. Interestingly, there is an overall relationship between seed size, seed nitrogen content and nodulation in legumes (Corby *et al*. 2011). Considering the same seed weight, seed nitrogen concentration is higher in nodulating than in non-nodulating species (Corby *et al*. 2011). As mentioned above, increased seed quality could have a positive effect on seed dispersal (Jordano 2013) and seedling recruitment (Leishman and Westoby 1994), but to our knowledge the effect of nodulation on both processes is yet unexplored. Additionally, different rhizobial strains within nodulating species might diverge on their effect on plant growth and reproduction (Parker 1995), which has been shown to occur for both native and invasive legumes (Thrall *et al*. 2007; Rodríguez-Echeverría *et al*. 2012). The variation due to different rhizobia in terms of plant reproduction and pollination, which might have relevant consequences for plant invasion, remains to be tested.

## Influence of Below–Aboveground Links on Alien Plant Invasions

Alien plants that do not depend on mutualists are more likely to become naturalized and invasive in new geographic ranges (Richardson *et al*. 2000). Accordingly, many alien invasive plants do not associate with mycorrhizal fungi or form a flexible facultative interaction (Pringle *et al*. 2009*b*), which allows the evolution towards a lower dependence on mycorrhizal fungi in the new range (Seifert *et al*. 2009). Those alien invaders that do rely on soil mutualists are mostly generalists whereas only a few described invasive plant species associate exclusively with specific mycorrhizal species (Pringle *et al*. 2009*a*, *b*; Moora *et al*. 2011). In fact, having a narrow range of symbiotic partners can impair the invasion of alien plants, such as Pinaceae species in the Southern Hemisphere, since they need the co-introduction and expansion of their mutualists to spread outside plantations (Nuñez *et al*. 2009). The same pattern is observed on aboveground mutualisms. Most invasive alien plants are generalists both for pollination and for seed dispersal (Traveset and Richardson 2006), so they are easily integrated into pollination and dispersal communities. In fact, alien species that do not become naturalized and invasive have a lower capacity to attract pollinators in the new geographical range (Razanajatovo *et al*. 2015).

**Figure 1.**
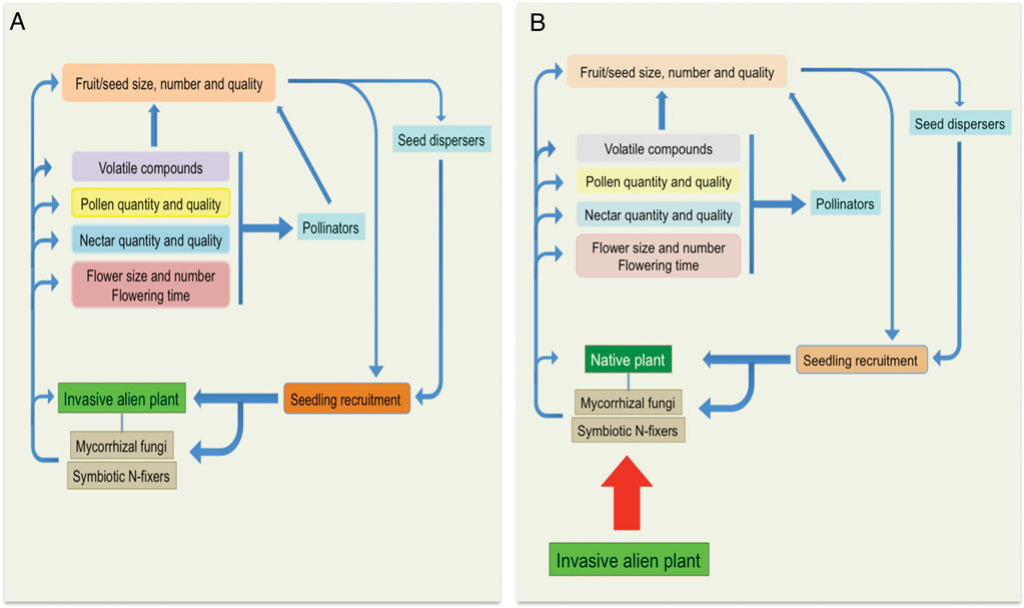
Different effects of invasive alien plants on above–belowground links depending on their association with soil mutualists. (A) Alien species with effective belowground mutualisms would benefit from positive links between those and aboveground mutualists resulting in a higher reproduction success and positive feedback on soil microbes. (B) Alien species that do not rely on belowground mutualists lead to decreases in microbial abundance, which affect native species and their interactions with pollinators and seed dispersers.

There is much evidence showing how below and aboveground mutualisms, separately, can enhance plant invasions (reviewed in Traveset and Richardson 2014). On the contrary, nothing is known about the extent to which interactions between the two types of mutualisms influence the population density of the invasive plant and the progress of invasion. So far, we only have indirect evidence on different aspects of such influence. For instance, we do know that some reproductive traits (flower size, amount of flowers, nectar quality, fruit display etc.) are associated with invasiveness (e.g. Pyšek and Richardson 2007). Hence, since belowground mutualists can have a positive effect on plant reproductive traits, we hypothesize that invasive species able to associate with effective soil mutualists in the introduced area would have an advantage over those species that cannot find adequate mutualists (Fig. 1).

In turn, we also hypothesize that the spreading of an invasive plant could modify the interaction between below- and aboveground mutualists. Indirect support for this is also available. Modifications in both AM fungi abundance and community composition have been observed in areas invaded by alien plants (e.g. Hawkes *et al*. 2006). Thus, invasive plants can establish positive feedbacks with the ‘new mycorrhizal community’ that have the potential of influencing reproductive traits differently. Changes in the mycorrhizal community introduced by invasive alien plants also have the potential to alter interactions of mycorrhizal fungi with native plants (Fig. 1). Some invasive plant species harbour more diverse AM fungal communities than native co-occurring plants without introducing alien fungi (Lekberg *et al*. 2013). This increase in AM fungal richness happens when the alien invader is a better mycorrhizal host than native plants, and has been documented for forbs invading grass-dominated communities (Lekberg *et al*. 2013). In most cases, however, alien plants seem to have a detrimental effect on native mycorrhizal fungi, particularly when alien species are non-mycorrhizal, reducing spore abundance, viability and infectivity and reducing fungal richness and diversity (e.g. Stinson *et al*. 2006; Callaway *et al*. 2008; Wolfe *et al*. 2008; Pringle *et al*. 2009*b*; Vogelsang and Bever 2009; Cantor *et al*. 2011; Meinhardt and Gehring 2012). Native plant species growth and survival are also affected by these changes in mycorrhizal communities (Callaway *et al*. 2008). Thus, we hypothesize that the reproductive traits of mycorrhizal native plants are likely to be affected as well by the new mycorrhizal communities found in invaded soils, which might in turn influence their population dynamics and the invasion process (Fig. 1).

Alien plants can also boost the expansion and invasion of associated alien microorganisms. Many alien microorganisms are usually unintentionally co-introduced with forestry tree seedlings or potting medium (Jairus *et al*. 2011). To establish in novel habitats and maintain viable soil populations, such microorganisms can face strong challenges, including abiotic stresses in the soil, competition with other soil biota and securing access to hosts at adequate densities (Porter *et al*. 2011). However, once introduced, alien microorganisms may also shift hosts and become naturalized or invasive in the introduced range. For example, alien nodulating bacteria introduced with invasive Australian *Acacia* species are effective colonizers of the roots of co-occurring native legumes and can displace native microsymbionts (Rodríguez-Echeverría 2010; Rodríguez-Echeverría *et al*. 2011; Ndlovu *et al.* 2013). However, the symbiosis between alien rhizobia and native plants is not always effective for native plants (Rodríguez-Echeverría *et al*. 2012) and, thus, it might affect aboveground mutualisms. Several species of ectomycorrhizal (EM) fungi that have been moved to new ranges are also known to have shifted hosts (e.g. Díez 2005; Pringle *et al*. 2009*a*; Vellinga *et al*. 2009). These EM fungi can associate with native trees and shrubs in the areas of introduction, although apparently without a reduction in the diversity of native EM fungi (Wolfe *et al*. 2010; Nuñez and Dickie 2014). It will be interesting to assess whether and how such novel plant–fungal interactions influence pollination and seed dispersal of native trees and shrubs and whether they differ from those established between native plant–fungal interactions. Based on previous studies, we hypothesize that novel interactions might not be as effective at initial stages of invasion stages as those interactions that have coevolved together (Rodríguez-Echeverría *et al*. 2012).

A phenomenon being increasingly reported is that of invasional meltdown (Simberloff and Von Holle 1999; but see Simberloff 2006) by which alien plants and alien mutualists enhance each other's invasions. There are many examples of invasional meltdowns involving alien plants and alien pollinators (Simberloff and Von Holle 1999 and references therein), seed dispersers (Bourgeois *et al*. 2005), mycorrhizal fungi (Dickie *et al*. 2010) and rhizobia (Rodríguez-Echeverría 2010). An interesting case of invasional meltdown including alien mycorrhizal fungi, plants and aboveground dispersers has been recently documented (Nuñez *et al*. 2013). These authors have shown that alien deer and boar consume fruiting fungal bodies in Pinaceae plantations in Argentina and can disperse EM fungal spores up to distances of 12 km. Since the naturalization and invasion of Pinaceae in the Southern Hemisphere is limited by the lack of compatible EM fungi outside plantations (Nuñez *et al*. 2009), alien mammals would thus facilitate the establishment of wind-dispersed Pinaceae seeds far from the plantation through the dispersal of EM fungi. We hypothesize that unexplored similar links between below- and aboveground mutualists could be contributing to the spread and invasion of other alien plant species.

## Future Research Avenues

We propose some topics in which research could be fruitful providing new insights into the role of biotic interactions in the invasion by alien plants. We have organized them from topics that are currently understudied to more complex studies on the ecological and evolutionary consequences of the interactions considered in this review.

### Changes in native soil microbial communities by invasive plants and reproduction of native plants

Alien plant species can alter or reduce soil microbial communities through changes in litter quantity and quality and the release of allelopathic compounds (e.g. Lorenzo *et al*. 2013). These changes can lead to the establishment of positive plant–soil feedbacks that promote invasion. Additionally, the interaction between native plants and these altered soil microbial communities can also affect the establishment and growth of native plants (e.g. Rodríguez-Echeverría *et al*. 2013). However, it remains unknown whether such novel plant–soil interactions can influence pollination and seed dispersal of native plant species in different ways than native plant–soil interactions.

### Interactions with seed dispersers

Belowground mutualists can increase the number, size or nutrient content of seeds, which usually result in a higher probability of seedling survival. Whether native and alien seed dispersers can detect changes in fruit and seed quality due to belowground mutualisms is still unknown, but certainly worth exploring. Such interaction will link belowground mutualisms with the ability of alien plants to disperse to new areas and become invasive.

### Invasional meltdowns involving below- and aboveground interactions

The co-invasion by alien plants and alien pollinators has been described for alien honeybee and bumblebee species pollinating invasive plants (Traveset and Richardson 2014). This mechanism could be more complex if we take into account that soil mutualistic microorganisms alter reproductive plant traits in ways that increase the production of flowers and pollen. A higher availability of resources may promote more frequent interactions with invasive pollinators that are usually social insects with high energetic demands. There is less evidence for plant–insect interactions helping invasive microorganisms establishing in new areas, although novel plant–microbe–insect associations could provide new paths for the spread of microbial pathogens (Bennett 2013; Kempel *et al*. 2013; Traveset and Richardson 2014). Other type of invasional meltdowns that have begun to be described recently are those between alien ungulates and mycorrhizal fungi that result in the spread of alien plants and/or the suppression of natives. Alien vertebrates can eat and disperse alien mycorrhizal fungi that are obligatory symbionts of alien *Pinus* species, thus, allowing the establishment of the symbiosis outside the introduction area (Nuñez and Dickie 2014). Also, the negative effect of invasive ungulates on native plant species is mediated, at least on some cases, by their effect on AM fungi (Kardol *et al*. 2014). Changes in abiotic soil properties, particularly in soil bulk density, induced by invasive ungulates have a negative effect on mycorrhizal colonization that results in a reduction of seedling growth (Wardle *et al*. 2001; Kardol *et al*. 2014).

### Mycorrhizal succession and reproduction of alien invasive plants

The community of mycorrhizal fungi colonizing woody plants undergoes unidirectional changes during host growth (e.g. Twieg *et al*. 2007). An interesting unexplored question is whether the start of sexual reproduction in woody plants matches specific changes in the composition of their associated mycorrhizal communities. Since alien woody invasive plants have a significantly shorter juvenile period (Rejmánek and Richardson 1996), it would be interesting to know whether this trait is related to the mycorrhizal status of the invader and has any effect on the composition of the associated mycorrhizal community.

### Landscape variability in below- and aboveground mutualisms

The spatial and temporal heterogeneity in abiotic conditions should be taken into account when studying the importance of below–aboveground mutualisms links for alien plant invasions. This would allow us to have a better understanding of the importance of below–aboveground linkages between mutualists for plant invasions. Soil heterogeneity in nutrients and mutualists might be coupled with pollinator and disperser landscapes but studies at landscape level on either belowground or aboveground mutualists and invasive plants are very scarce. In a recent study, however, Bennett and Strauss (2013) have shown that alien species are less responsive than native species to landscape variability in soil communities. The heterogeneity in abiotic conditions across environments can actually generate mosaics in the outcome of the mutualism for either partner, influencing the role of mutualisms during invasion. Given the considerable carbon costs of supporting soil mutualists (Vitousek *et al*. 2002; Olsson *et al*. 2010), these associations would be advantageous for invasive plants only in specific contexts (Porter *et al*. 2011) and might be mediated by other biotic interactions like pollination or seed dispersal. If soil mutualisms can modify reproductive traits to attract more aboveground mutualists, this would increase plant reproduction success and contribute to the maintenance of the mutualism, even if costly for the plant.

### Shifts in evolutionary trajectories

Alien plants might establish novel interactions with resident mutualists that can lead to rapid changes in the evolutionary trajectories of both partners. The introduction to a new region implies loosing interacting species, which might lead to (i) a failure in establishment and, therefore, naturalization and invasion of the alien species or (ii) the evolution of reduced dependence on mutualisms for successful invaders. Rapid evolution of alien plants towards a loss of dependency on belowground mutualists has been detected during invasion (e.g. Seifert *et al*. 2009) but similar studies for aboveground mutualists are scarce (but see Correia *et al*. 2014). For successful invaders, host shifts and the presence of dense populations of alien plants might result in the evolution of more generalist mutualisms in invaded areas (Kiers *et al*. 2010), although these processes are likely to be context-dependent.

## Conclusions

In spite of the growing literature on below–aboveground links and how these affect ecosystem structure and dynamics, more research is needed to understand their relevance during invasion. Studies on ecological interactions and community ecology provide evidence for the existence of links between below- and aboveground mutualists. We propose that these links should be incorporated into the mechanisms driving the invasion by alien plants. Many invaders do not establish mutualisms with mycorrhizal fungi or rhizobia, but those that do so provide interesting case studies for the ecology and evolution of above–belowground links. Mycorrhizal fungi, and to a lesser extent rhizobial partners, can alter plant resource allocation to reproductive structures. In general, mycorrhizas have a positive effect on the number of flowers as well as the quantity and quality of pollen and nectar. These changes have a subsequent positive effect on the interaction with pollinators (which might also be native or alien), and might result in higher fruit and seed set. It would be expected that all these changes also modify the interaction with seed dispersers in ways that benefit the alien plant. Thus, differences in the availability of belowground mutualists might affect alien plant species establishment, reproduction and dispersal. These links can also have consequences for the populations of interacting aboveground mutualists and their association with native plants. In this sense, invasive plants that are not mycorrhizal can have a negative effect on native mycorrhizal fungal communities and, thus, could also affect the interaction between native plants and aboveground mutualists (e.g. Stinson *et al*. 2006; Wolfe *et al*. 2008).

## Sources of Funding

This work was partially supported by the project MUTUALNET (PTDC/BIA-BEC/103507/2008) funded by the Portuguese Foundation for Science and Technology (FCT). The FCT and European Social Fund financed the work of S.R.E. (development grant IF/00462/2013).

## Contributions by the Authors

Both authors conceived the idea of the manuscript. S.R.E. wrote the main text and A.T. reviewed and completed the final version. Both authors contributed to the figures and tables.

## Conflict of Interest Statement

None declared.
